# Significantly increased load of hereditary cancer-linked germline variants in infertile men

**DOI:** 10.1093/hropen/hoaf008

**Published:** 2025-02-21

**Authors:** Anu Valkna, Anna-Grete Juchnewitsch, Lisanna Põlluaas, Kristiina Lillepea, Stanislav Tjagur, Avirup Dutta, Kristjan Pomm, Margus Punab, Maris Laan

**Affiliations:** Chair of Human Genetics, Institute of Biomedicine and Translational Medicine, University of Tartu, Tartu, Estonia; Chair of Human Genetics, Institute of Biomedicine and Translational Medicine, University of Tartu, Tartu, Estonia; Chair of Human Genetics, Institute of Biomedicine and Translational Medicine, University of Tartu, Tartu, Estonia; Chair of Human Genetics, Institute of Biomedicine and Translational Medicine, University of Tartu, Tartu, Estonia; Andrology Clinic, Tartu University Hospital, Tartu, Estonia; Chair of Human Genetics, Institute of Biomedicine and Translational Medicine, University of Tartu, Tartu, Estonia; Andrology Clinic, Tartu University Hospital, Tartu, Estonia; Chair of Human Genetics, Institute of Biomedicine and Translational Medicine, University of Tartu, Tartu, Estonia; Andrology Clinic, Tartu University Hospital, Tartu, Estonia; Department of Surgery, Institute of Clinical Medicine, University of Tartu, Tartu, Estonia; Chair of Human Genetics, Institute of Biomedicine and Translational Medicine, University of Tartu, Tartu, Estonia

**Keywords:** male infertility, hereditary cancer, germline variants, molecular diagnosis, exome sequencing, shared genetic etiology, BRCA2, Fanconi anemia pathway, multidisciplinary management

## Abstract

**STUDY QUESTION:**

What is the load and profile of hereditary cancer-linked germline variants in infertile compared to fertile men?

**SUMMARY ANSWER:**

This study showed almost 5-fold enrichment of disease-causing findings in hereditary cancer genes in infertile compared to fertile men (6.9% vs 1.5%, *P *=* *2.3 × 10^−4^).

**WHAT IS KNOWN ALREADY:**

Epidemiological studies have revealed that men with low sperm count have a 2-fold higher risk of developing cancer during their lifetime. Our recent study observed a 4-fold increased prevalence of cancer in men with monogenic infertility compared to the general male population (8% vs 2%). Shared molecular etiologies of male infertility and cancer have been proposed.

**STUDY DESIGN, SIZE, DURATION:**

This retrospective study analyzed germline likely pathogenic and pathogenic (LP/P) variants in 157 hereditary cancer genes in 522 infertile and 323 fertile men recruited to the ESTonian ANDrology (ESTAND) cohort.

**PARTICIPANTS/MATERIALS, SETTING, METHODS:**

All study participants (n = 845) had been recruited and phenotyped at an Andrology Clinic. Identification of LP/P variants in the cancer gene panel was performed from an exome sequencing dataset generated for the study cohort. All variants passed an automated filtering process, final manual assessment of pathogenicity, and experimental confirmation using Sanger sequencing. Retrospective general health records were available for 36 out of 41 (88%) men with LP/P findings.

**MAIN RESULTS AND THE ROLE OF CHANCE:**

Infertile men presented a nearly 5-fold higher load of LP/P findings (36 of 522 cases, 6.9%) compared to fertile subjects (5 of 323, 1.5%; odds ratio (OR) = 4.7, 95% CI 1.81–15.5; *P* = 2.3 × 10^−4^) spanning over 24 hereditary cancer genes. The prevalence of findings was not significantly different between azoospermic and oligozoospermic cases. There was also no enrichment of findings in men with a history of cryptorchidism. By the time of the study, six men carrying hereditary cancer variants had been diagnosed with a tumor. Family members affected with cancer had been documented for 10 of 14 cases with available pedigree health data.

Nearly half of the infertile men with LP/P findings (17 out of 36) carried variants in genes belonging to the Fanconi anemia (FA) pathway involved in the maintenance of genomic integrity in mitosis and meiosis, repair of DNA double-stranded breaks, and interstrand crosslinks. Overall, FA-pathway genes *BRCA2* (monoallelic) and *FANCM* (biallelic) were the most frequently affected loci (five subjects per gene).

LP/P findings in pleiotropic genes linked to human development and hereditary cancer (*TSC1*, *PHOX2B*, *WT1*, *SPRED1*, *NF1*, *LZTR1*, *HOXB13*) were identified in several patients with syndromic phenotypes. Four cryptorchid infertile men were carriers of *MLH1*, *MSH2*, and *MSH6* variants implicated in Lynch syndrome. Future studies will reveal whether this observation is a by chance or replicable finding.

Most hereditary cancer genes with LP/P variants show high expression in one or more testicular cell types, and mouse models for 15 of 24 affected genes have been reported to exhibit male sub- or infertility. These data support shared genetic etiology of impaired spermatogenesis and cancer. A significantly increased fraction of cancer-linked variants in infertile compared to fertile men could also explain the reported high prevalence of cancer as a comorbidity in male infertility.

**LARGE SCALE DATA:**

All hereditary cancer-linked variants identified in this study have been submitted to the National Center for Biotechnology Information (NCBI) ClinVar database (https://www.ncbi.nlm.nih.gov/clinvar/).

**LIMITATIONS, REASONS FOR CAUTION:**

All recruited participants were of white European ancestry and living in Estonia. Thus, the results might not apply to other ethnic groups. Due to the young age of study participants (median age 34.4 years), the true incidence of cancer during lifetime could not be assessed. As retrospective clinical data were not available for all men, it was not possible to evaluate all possible genotype–phenotype links. The absence of genetic data from family members precluded the assessment of the hereditary nature of the variants or their potential *de novo* occurrence.

**WIDER IMPLICATIONS OF THE FINDINGS:**

Infertility affects about 7–10% of men worldwide. In this study, one in 15 men with spermatogenic failure carried germline LP/P variants in hereditary cancer genes. As exome sequencing is gradually entering the molecular diagnostics setup in andrology, analyzing hereditary cancer-linked variants in the workup of infertile men will offer additional clinical benefits. Male factor infertility is typically diagnosed in men in their 30s, often before the onset of cancer or its symptoms. Early knowledge of germline predisposition to cancer enables timely screening and multidisciplinary management options, potentially improving the prognosis. The study data provide support for the shared monogenic etiologies of hereditary cancer and spermatogenic failure.

**STUDY FUNDING/COMPETING INTEREST(S):**

This study was funded by the Estonian Research Council grant PRG1021 (M.L. and M.P.). The authors declare no conflicts of interest.

WHAT DOES THIS MEAN FOR PATIENTS? Every 10th man suffers from sub- or infertility. Previous studies have shown that men with low sperm counts are twice as likely to develop cancer during their lifetime. We investigated whether these observations may be explained by genetic predisposition. The study found genetic variants implicated in hereditary cancer syndromes in every 15th infertile man compared to one in 64 fertile subjects. This translates to a nearly 5-fold higher risk for early-onset cancers in men with low sperm counts. The study outcomes have immediate clinical implications as men are typically seeking infertility management in their 30s when they are asymptomatic for cancer on most occasions. Including testing of hereditary cancer-linked variants in the genetic workup of infertile men will provide a significant added value, enabling timely counseling and management of reproductive and general health strategies.

## Introduction

Male factor infertility affects 7–10% of men worldwide (Agarwal *et al.*, 2021), and it has been suggested as an independent risk factor for a range of chronic diseases (Punab *et al.*, 2017; Del Giudice *et al.*, 2020). Epidemiological studies have revealed that men with low sperm count have a 2-fold higher risk for cancers (Eisenberg *et al.*, 2013; Ramsay *et al.*, 2024). A recent study observed a 4-fold increased prevalence of cancer in men with monogenic infertility compared to the general male population (8% vs 2%) (Lillepea *et al.*, 2024). Two scenarios could explain these observations. One possibility is that infertility and testicular malfunction will predispose to an overall suboptimal male physiology and, therefore, to a higher risk of chronic diseases, including cancer. However, other studies have shown an increased risk of multiple types of cancer among relatives of men with fertility problems (Ramsay *et al.*, 2024). Thus, an alternative option is that impaired or failed sperm production and tumor development have joint contributing factors, and among these, overlapping genetic etiology represents an attractive scenario. Dual roles of pleiotropic genes responsible for DNA replication and repair, genome integrity, and meiotic recombination have been suggested, supported by several mouse models presenting sub- or infertility (Nagirnaja *et al.*, 2018). Several genes critical in repairing DNA double-strand breaks (DSB) have been linked to hereditary cancer syndromes and infertility in both men and women (e.g. *BRCA2*, *FANCM*, *XRCC2*) (Kasak *et al.*, 2018; Tsui and Crismani, 2019).

We hypothesized that infertile men carry an increased load of hereditary cancer-linked germline variants compared to fertile men. Our pilot study analyzing 26 cancer genes in 836 idiopathic non-obstructive azoospermia (NOA) cases identified clinically valid findings in 1.6% of patients (Kasak *et al.*, 2022). The current study expanded the panel of hereditary cancer genes nearly 6-fold, including 157 loci. The main objective was to analyze the load and profile of hereditary cancer-linked germline variants in 522 infertile compared to 323 fertile men.

## Materials and methods

### Ethics statement

The study was approved by the Ethics Review Committee of Human Research of the University of Tartu, Estonia (permission no. 74/54 and 118/69 with last amendment 288/M-13). Written informed consent was obtained from each patient before recruitment to evaluate and use their clinical data for scientific purposes. The study was carried out in compliance with the Helsinki Declaration.

### Study group formation, phenotyping, and testing for genetic infertility

All 845 study participants were recruited to the ESTonian ANDrology (ESTAND) cohort (Punab *et al.*, 2017), and the clinical data and biological samples were collected at the Andrology Clinic of Tartu University Hospital (AC-TUH). All participants were of white European ancestry and living in Estonia. An overview of the study design and flow is shown in Supplementary Fig. S1.

The study analyzed 522 men with idiopathic infertility (median age 34.4 years) and 323 fertile controls (31.0 years). The study group formation has been recently described in detail (Juchnewitsch *et al.*, 2024; Lillepea *et al.*, 2024). Male factor infertility (total sperm count ≤39 million per ejaculate) was defined based on World Health Organization (WHO) guidelines (World Health Organization, 2021). Cryptorchidism refers to at least one testicle missing in the scrotum at the recruitment or medical history of cryptorchidism resolved by orchidopexy or spontaneous descent. Known non-genetic and genetic factors affecting spermatogenesis were excluded (e.g. hypogonadism, seminal tract obstruction, sexual dysfunction, androgen abuse, severe traumas, and operations in the genital area, including vasectomies, chemo- or radiotherapy, cytogenetic abnormalities, and Y-chromosome microdeletions) (Punab *et al.*, 2017; World Health Organization, 2021). No disease-causing variants were identified in the *CFTR* (MIM: 602421; autosomal recessive) and *ADGRG2* (MIM: 300572; X-linked) genes, excluding cases of obstructive azoospermia. Analysis of 660 male infertility candidate genes identified a monogenic cause for infertility in 12.7% and oligogenic infertility in 1.3% of patients in the study group (Juchnewitsch *et al.*, 2024; Lillepea *et al.*, 2024).

The control group was recruited among normozoospermic partners of pregnant women (Ehala-Aleksejev and Punab, 2015; Punab *et al.*, 2017). Physical examination, genital, and testicular phenotyping were documented for all 845 subjects by andrology specialists at AC-TUH (Supplementary Materials and Methods). Sperm and hormonal analysis from blood samples were conducted in the United Laboratory of Tartu University Hospital (Supplementary Table S1).

None of the subjects analyzed in this study had been preselected for their medical history or familial predisposition to malignancies. For 36 of 41 patients with cancer-linked findings, retrospective general health data were collected. Among these, pedigree health data for 14 infertile men had also been documented. For fertile men with findings, the medical history of family members was unavailable.

### Candidate gene panel

**Figure 1.**
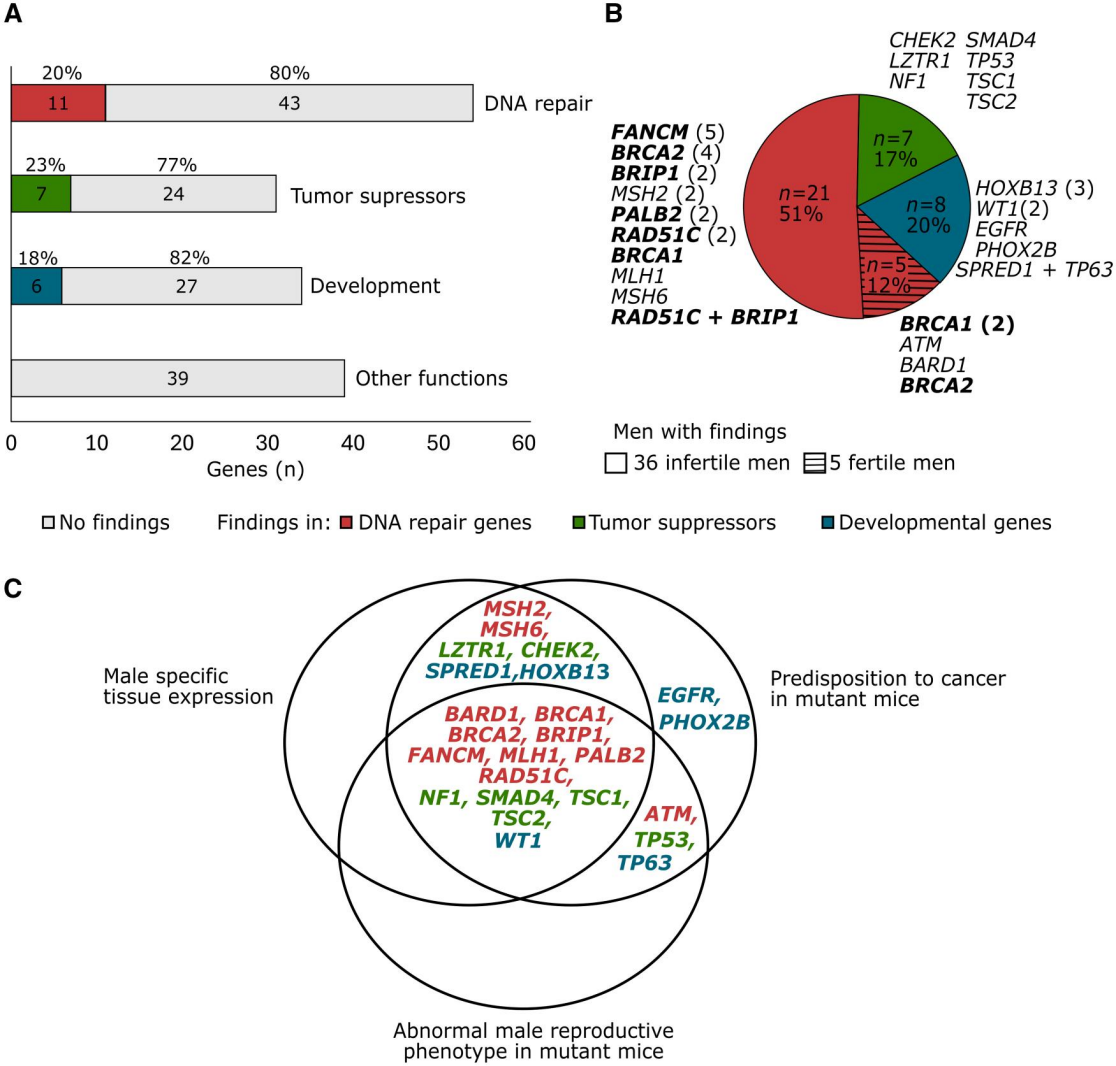
**Molecular functions of hereditary cancer genes with likely pathogenic or pathogenic (LP/P) variants identified in infertile and fertile men**. (**A**) Number and proportion of genes with findings in each functional category among 157 analyzed loci. (**B**) Genes with LP/P findings stratified based on their molecular functions. The number of affected subjects (total, n = 41) is marked in brackets next to the gene. Genes within the Fanconi anemia pathway are highlighted in bold. (**C**) Literature data of human testicular gene expression and mutant mouse models for genes with findings. Details are presented in Supplementary Table S7. LP, likely pathogenic; P, pathogenic.

The panel of 157 genes linked to hereditary cancer syndromes comprised 113 loci from the Illumina TruSight Hereditary Cancer Panel (https://www.illumina.com/products/by-type/clinical-research-products/trusight-cancer-hereditary.html), 10 additional genes linked to monogenic cancers in the Online Mendelian Inheritance in Man (OMIM) database (https://www.omim.org/), and 34 genes in the Catalogue Of Somatic Mutations In Cancer (COSMIC) Cancer Gene Census (https://cancer.sanger.ac.uk/census) reported to be involved in hereditary cancers (Supplementary Table S2). Genes were categorized into four subgroups based on their molecular function: DNA repair (n = 54), tumor suppressors (n = 31), development (n = 33), and other functions (n = 39) (Fig. 1A).

Approximately half of the genes (85 of 157) were linked to autosomal dominant (AD) forms of hereditary cancers, followed by 45 autosomal recessive (AR) cancer genes. Various malignancies with either monoallelic or biallelic inheritance mode (AD/AR) have been linked to 23 hereditary cancer genes, and four loci are categorized as X-linked.

### Data generation by whole-exome sequencing, quality control, and variant annotation

Whole-exome sequencing (WES) library preparation and data generation by next-generation sequencing (NGS) service laboratories have been described in detail (Juchnewitsch *et al.*, 2024; Lillepea *et al.*, 2024) (Supplementary Materials and Methods). In all study subjects, WES was undertaken using genomic DNA extracted from the whole blood samples. Raw sequencing reads were aligned to the GRCh38 human genome assembly. The Variant Call Format (VCF) files of all samples were filtered for variant quality (exclusion criteria: depth of coverage <10 and genotype quality <20) individually and merged into a single VCF file that was further segmented into 24 individual chromosome files (chr1-22, X, and Y) for variant annotation. Merging, filtering, and splitting of VCF files was performed with bcftools (v1.14; Danecek *et al.*, 2021). VCF files were annotated with Ensembl Variant Effect Predictor (VEP, v105; McLaren *et al.*, 2016) in the offline mode using a predetermined set of flags and plugins as indicated in Supplementary Table S3.

### Variant filtering and pathogenicity assessment

All variants in the VEP output files were subjected to several stages of variant filtering according to the developed in-house pipeline (Juchnewitsch *et al.*, 2024; Lillepea *et al.*, 2024). Detailed variant filtering criteria are presented in Supplementary Table S4. Variants reported as confidently pathogenic (P) and/or likely pathogenic (LP) in the National Center for Biotechnology Information (NCBI) ClinVar database (Landrum *et al.*, 2018) were automatically retrieved from the VEP output file (column ‘clinvar_clnsig’), and no additional filters were applied. For the rest of the variants, minor allele frequency (MAF) of 0.5% in the general population (gnomAD v4.0.0; https://gnomad.broadinstitute.org) and Combined Annotation Dependent Depletion (CADD) score ≥20 (Rentzsch *et al.*, 2019) were used as cutoffs to filter potential disease-causing variants for further assessment (Supplementary Materials and Methods). Global and European MAF data of identified variants was retrieved from the gnomAD database (version 4.0.0, https://gnomad.broadinstitute.org). Singleton heterozygous variants in genes reported in only AR forms of hereditary cancer were excluded from further analysis.

The AI-based platform Franklin by Genoox (https://franklin.genoox.com) and data submitted by other teams to the NCBI ClinVar database (Landrum *et al.*, 2018) were used to shortlist the most likely candidate variants with disease-causing effects. Variants predicted to be unanimously likely benign (LB) or benign (B) were excluded. All retained variants passed a visual inspection for the quality of sequencing reads using the Integrative Genomics Viewer (IGV) software (Robinson *et al.*, 2023), and low-confidence variant calls were discarded. Following the recommendations of the American College of Medical Genetics and Genomics (ACMG) (Richards *et al.*, 2015), a manual pathogenicity assessment was carried out in parallel by two researchers. The final pathogenicity assessment also included recent literature, database records, and clinical data collected during this study.

All LP/P variants were confirmed by Sanger sequencing. PCR primers are available in Supplementary Table S5, and chromatograms of validated variants are shown in Supplementary Fig. S2.

## Results

### Significantly higher load of findings in hereditary cancer genes in infertile vs fertile men

A panel of 157 hereditary cancer genes was analyzed in 845 ESTAND cohort participants, including 522 infertile and 323 fertile men (Table 1). As a result, 40 variants were classified as LP or P, distributed among 24 of 157 analyzed genes (15.3%) (Fig. 1A, Supplementary Table S6). Most of these variants (28 of 40, 70%) have been previously reported in the NCBI ClinVar database as LP or P, including nine pathogenic variants reviewed by expert panels (Table 2). Disease-causing variants were found in 41 subjects, including loss-of-function (LoF: nonsense, frameshift, or splice-site changes) variants in 24 and missense substitutions in 18 men. Among them, two men carried digenic findings. Most detected LP/P variants were linked to AD forms of cancers.

**Table 1.** Clinical parameters of the study subjects stratified by the analyzed subgroups.

Parameter	Infertile men (n = 522)	Fertile men (n = 323)
Age (years)	34.4 (22.9–52.2)*	31.0 (23.0–45.0)
Height (cm)	180.0 (169.0–193.0)	180.0 (171.0–191.0)
Weight (kg)	86.0 (64.8–117.4)*	82.3 (64.0–107.9)
BMI (kg/m^2^)	26.5 (20.4–36.3)*	25.0 (20.0–32.3)
FSH (IU/l)	18.8 (5.2–50.5)	3.5 (1.4–8.2)
LH (IU/l)	8.6 (3.3–20.5)	3.6 (1.5–6.7)
Testosterone (nmol/l)	14.4 (5.9–28.6)	16.1 (7.9–26.9)
PSA (µg/l)	0.6 (0.2–1.6)	0.7 (0.3–1.4)
Total sperm count (×10^6^/ejaculate)	0 (0–8.4)	303.1 (70.3–978.2)
Total testis volume (ml)	27.0 (9.0–46.0)	46.0 (34.0–63.0)
Cryptorchidism (n, %)	156 (29.9%)	8 (2.5%)

Median values (5–95%) are presented unless stated otherwise. Reference values are presented in Supplementary Table S1.

* Significant difference (Mann–Whitney *U* test, *P* < 10^−3^) in the parameter not defined by the study group formation criteria.

PSA, prostate-specific antigen.

**Table 2.** Clinical information on the study subjects with (likely) pathogenic findings in the analyzed 157 genes.

	Variant data (hg38)	Clinical data							Pathogenicity classification	
No. (age)	Gene: cDNA (protein change) rs–number; MAF	H/W (cm/kg) BMI	Spc ×10^6^/ej.	TV (ml) Sin+dex total	FSH LH (IU/l)	T (nmol/l)	PSA (µg/l)	Medical history of cancer and congenital anomalies	Franklin ClinVar	Final
1 (22 y)	*ATM:* c.8565T>G (p.Ser2855Arg) rs780905851; 2.1 × 10^–6^	178/77 24.3	270.0 (fertile)	23 + 23 46	9.0 4.9	16.6	0.7	Not reported	P P, LP^§^	LP
2 (31 y)	*BARD1:* c.2300_2301del (p.Val767fs) rs750413473; 2.5 × 10^–5^	177/81 25.8	348.9 (fertile)	22 + 22 44	5.5 6.7	17.7	0.8	Not reported	P P, LP^§^	LP
3 (30 y)	*BRCA1*: c.5266dup (p.Gln1756fs) rs80357906; 6.9 × 10^–5^	170/67 23.3	524.2 (fertile)	24 + 25 49	2.6 1.4	9.9	n.a.	n.a.	P P^#^	P
4 (31 y)	*BRCA1:* c.5117G>A (p.Gly1706Glu) rs80356860; 6.8 × 10^–7^	177/90 28.9	3.1	18 + 14 32	27.5 9.4	22.2	1.4	Early-onset breast cancer (mother)*	P P^#^	P
5 (39 y)	*BRCA1*: c.4035del (p.Glu1346fs) rs80357711; 8.2 × 10^–6^	180/98 30.2	363.8 (fertile)	30 + 30 60	6.2 5.2	16.5	n.a.	n.a.	P P^#^	P
6^a^ (31 y)	*BRCA2*: c.1796_1800del (p.Ser599Ter) rs276174813; 2.8 × 10^–6^	176/70 22.7	0	22 + 25 47	5.7 5.6	14.7	0.4	Early-onset thigh leiomyosarcoma (patient)	P P^#^	P
7^a^ (34 y)	*BRCA2:* c.3646_3649dup (p.Arg1217fs); n.a.	173/78 26.1	0	17 + 16 33	24.9 3.7	12.8	n.a.	Not reported	LP LP	LP
8 (41 y)	*BRCA2:* c.3847_3848del (p.Val1283fs) rs80359405; 3.7 × 10^–5^	186/101 29.3	10.3	10 + 12 22	20.0 5.6	8.6	0.7	Breast cancer (mother)*; prostate cancer (father)*	P P^#^	P
9 (28 y)	*BRCA2:* c.5351dup (p.Asn1784fs) rs80359507; 2.7 × 10^–6^	190/74 20.4	0	12 + 12 24	25.8 12.7	22.4	0.6	Not reported	P P^#^	P
10 (31 y)	*BRCA2:* c.7879A>T (p.Ile2627Phe) rs80359014; 6.4 × 10^–6^	175/84 27.4	126.0 (fertile)	22 + 22 44	1.9 2.0	12.8	0.4	n.a.	P P^#^	P
11 (52 y)	*BRIP1:* c.806C>G (p.Ser269Ter) rs1412610651; 6.6 × 10^–6^	181/81 24.8	0	9 + 11 20	46.4 27.1	9.7	0.4	Not reported	P P, LP	LP
12^b^ (50 y)	*BRIP1:* c.139C>G (p.Pro47Ala) rs28903098; 3.7 × 10^–4^	181/104 31.7	0	5 + 5 10	13.7 8.4	3.2	0.7	Brain cancer (mother)*; gynecological cancer (sister)*; early-onset breast cancer (niece)*; gastric cancer (father)	VUS P, VUS, LB	LP
13 (44 y)	*CHEK2*: c.1169A>C (p.Tyr390Ser) rs200928781; 1.4 × 10^–5^	185/111 32.4	0	20 + 20 40 (b-CR)	12.6 2.2	12.2	0.3	n.a.	P P, LP	LP
14 (30y)	*EGFR:* c.2061 + 2T>C rs587777939; 5.5 × 10^–6^	186/85 24.7	0.4	13 + 16 29 (u-CR)	18.9 11.0	11.9	0.8	Not reported	P LP	LP
15^c^ (58 y)	*FANCM:* c.5101C>T (p.Gln1701Ter) rs147021911; 9.5 × 10^–4^ (homozygous)	180/85 26.1	0	9 + 10 19	42.3 18.6	5.8	0.3	Not reported	P P, LP	P
16^b^ (29 y)	*FANCM:* c.5101C>T (p.Gln1701Ter) rs147021911; 9.5 × 10^–4^ (homozygous)	174/70 23.1	0.3	14 + 15 29	33.1 4.5	15.6	n.a.	Early-onset prostate cancer (father)*	P P, LP	P
17^c^ (29 y)	*FANCM:* c.1491dup (p.Gln498fs) rs797045116; 2.5 × 10^–5^/ *FANCM:* c.4387–10A>G rs1555365959; n.a.	187/84 24.1	0	10 + 12 22	16.0 4.1	14.8	0.5	Not reported	P P, LP/ VUS P	LP/ LP
18^c^ (26 y)	*FANCM:* c.1491dup (p.Gln498fs) rs797045116; 2.46 × 10^–5^/ *FANCM:* c.4387–10A>G rs1555365959; n.a.	190/85 23.5	0	14 + 16 30 (u-CR)	31.0 9.6	14.1	0.4	Not reported	P P, LP/ VUS P	LP/ LP
19^b^ (28 y)	*FANCM:* c.1491dup (p.Gln498fs) rs797045116; 2.5 × 10^–5^ (homozygous)	185/79 23.1	0	13 + 13 26	17.9 10.5	10.9	n.a.	Not reported	P P, LP	P
20 (48 y)	*HOXB13*: c.650G>T (p.Arg217Leu) rs749518336; 1.3 × 10^–5^	173/85 28.3	0.3	12 + 17 29 (u-CR)	22.6 6.2	8.3	0.7	Not reported	LP VUS	LP
21 (39 y)	*HOXB13*: c.251G>A (p.Gly84Glu) rs138213197; 1.8 × 10^–3^	187/85 24.4	0	10 + 14 24	17.3 6.4	17.2	n.a.	Not reported	P P, LP	LP
22 (35y)	*HOXB13*: c.251G>A (p.Gly84Glu) rs138213197; 1.8 × 10^–3^	n.a.	0	9 + 9 18 (u-CR)	26.5 11.4	4.5	0.04	Klippel–Trenaunay–Weber syndrome (patient)	P P, LP	LP
23^d^ (38y)	*LZTR1:* c. 848 G > A (p. Arg283Gln) rs1223430276; n.a.	182/73 22	0	3 + 4 7 (u-CR)	62.0 10.2	17.8	n.a.	Schwannomatosis; RASopathy-linked conditions (patient)	LP P, LP	P
24 (32 y)	*MLH1*: c.74T>C (p.Ile25Thr) rs63750514; 2.7 × 10^–6^	186/105 30.5	0.5	24 + 0 24 (u-CR)	18.4 5.7	30.8	n.a.	Leukemia (mother’s half-sister)*	LP VUS	LP
25 (33 y)	*MSH2:* c.2039G>A (p.Arg680Gln) rs1203462814; 4.1 × 10^–6^	175/101 32.8	1.0	0 + 18 18 (u-CR)	13.9 7.6	12.3	0.4	Not reported	LP VUS	LP
26 (26 y)	*MSH2*: c.2048G>T (p.Gly683Val) rs755920849; 6.8 × 10^–6^	182/75 22.6	0	10 + 13 23 (b-CR)	32.6 11.5	11.9	n.a.	Not reported	LP VUS, LB	LP
27 (33 y)	*MSH6*: c.2569_2572del (p.Asp857fs) rs587779243; 1.4 × 10^–6^	184/94 28	34.7	21 + 0 21 (u-CR)	7.8 6.0	10.7	n.a.	Not reported	P P^#^	P
28^d^ (48 y)	*NF1*: c.4348G>T (p.Ala1450Ser) n.a.	177/90 28.8	8.4	8 + 12 20	36.3 9.8	12.2	2.6	RASopathy-linked conditions (patient); early-onset breast cancer (niece)*	P LP, VUS	LP
29 (26 y)	*PALB2:* c.2257C>T (p.Arg753Ter) rs180177110; 1.0 × 10^–5^	184/74 21.8	10.2	17 + 11 28 (u-CR)	27.0 7.0	16.6	0.8	n.a.	P P	P
30 (31 y)	*PALB2:* c.1592del (p.Leu531fs) rs180177102; 6.6 × 10^–5^	186/96 27.7	0.3	14 + 15 29	16.0 4.7	19.3	0.2	Not reported	P P^#^	P
31 (23 y)	*PHOX2B*: c.96del (p.Phe33fs) n.a.	183/93 27.9	0	8 + 10 18 (u-CR)	24.3 17.1	8.6	0.3	Hirschsprung disease; neoplasm of uncertain behavior (patient)	LP n.a.	LP
32 (32 y)	*RAD51C:* c.692C>G (p.Ser231Ter) rs1060502588; n.a.	176/84 27.1	0	23 + 23 46 (b-CR)	20.4 6.8	8.3	0.7	Not reported	P P	P
33 (30 y)	*RAD51C:* c.851_854dup (p.Met286fs) rs1060502605; n.a.	195/91 23.8	9.8	16 + 17 33	8.0 2.4	5.9	0.2	Not reported	P P, LP	P
34 (63 y)	*SMAD4:* c.983A>G (p.Tyr328Cys) n.a.	184/124 36.7	2.0	8 + 2 10	20.3 10.4	10.2	n.a.	Not reported	LP n.a.	LP
35^d^ (26 y)	*SPRED1* c.973C>T (p.Arg325Ter) rs1057518683; 1.2 × 10^–6^/ *TP63*: c.1283C>T (p.Pro428Leu) n.a.	193/101 27.1	6.3	25 + 25 50	15.2 12.9	23.3	1.0	Legius syndrome (patient, children); early-onset colon cancer (mother); cervical cancer (aunt)	P P/ LP LP	P/ LP
36 (29 y)	*TP53*: c.542G>A (p.Arg181His) rs397514495; 1.5 × 10^–5^	190/85 23.5	0.6	25 + 25 50	34.4 7.8	13.8	n.a.	Lymphoma (patient); prostate cancer (father)*; leukemia (grandfather)*	P P, LP, VUS	P
37 (35 y)	*TSC1*: c.1281del (p.Ala428fs) n.a.	185/102 29.8	1.4	16 + 13 29	18.4 10.9	11.2	0.6	Tuberous sclerosis, facial angiofibroma, ungual fibroma (patient); renal and gynecological cancer (grandmother)*	LP n.a.	LP
38 (43 y)	*TSC2*: c.2714G>A (p.Arg905Gln) rs45517259; n.a.	186/94 27.2	6.2	12 + 12 24	30.1 12.6	25.6	0.8	Not reported	P P, LP	P
39^b^ (32 y)	*WT1*: c.1244T>A (p.Met415Lys) n.a.	177/70 22.4	0	2 + 4 6 (b-CR)	49.9 26.9	9.3	0.3	Congenital anomaly of the left kidney (patient)	LP LP	LP
40^b^ (39 y)	*WT1*: c.451T>G (p.Trp151Gly) n.a.	177/86 27.5	0	8 + 10 18	29.7 5.6	11.2	n.a.	Basalioma; kidney complaints (patient) prostate cancer (grandfather)*; leukemia (uncle)*	LP LP	LP
41 (36 y)	*BRIP1*: c.2732dup (p.Thr912fs) rs752780954; 2.4 × 10^–5^/ *RAD51C*: c.1026 + 5_1026 + 7del rs587781410; 3.0 × 10^–5^	182/75 22.6	0	4 + 5 9 (b-CR)	79.4 40.3	7.8	0.1	Severe hypospadia (patient)	P P, LP/ LP P, LP	P/ LP

All variants are heterozygous unless indicated otherwise; minor allele frequency (MAF) was based on the gnomAD v4.0.0 database. Patient age, clinical, and andrological data are reported as gathered at recruitment. Open access platforms Franklin by Genoox (https://franklin.genoox.com/clinical-db/home) and NCBI ClinVar (https://www.ncbi.nlm.nih.gov/clinvar/) were utilized for the initial assessment of variant pathogenicity; the final variant classification used the ACMG guidelines (Richards *et al.*, 2015), also considering clinical data collected in this study. Further details are available in Supplementary Table S6.

ESTAND cases were originally reported in ^a^Kasak *et al.* (2022), ^b^Lillepea *et al.* (2024), ^c^Kasak *et al.* (2018) (cases 17 and 18 are brothers), ^d^Juchnewitsch *et al.* (2024).

* Genetic finding is linked to hereditary cancer type(s) reported in this family.

§ Heterozygous LP/P variants in these genes are mainly linked to breast cancer in females. Other cancer types are linked to biallelic LP/P variants.

# Reviewed as pathogenic by the NCBI ClinVar expert panel.

b-CR, bilateral cryptorchidism; dex, dexter (right); ej., ejaculate; H, height; LB, likely benign; LP, likely pathogenic; MAF, minor allele frequency; n.a., not available; No., case number; P, pathogenic; PSA, prostate-specific antigen; sin, sinister (left); Spc., total sperm count per ejaculate; T, testosterone; TV, testis volume; u-CR, unilateral cryptorchidism; VUS, variant of uncertain significance; W, weight; y, years.

Infertile men presented a nearly 5-fold higher load of LP/P variants (36 of 522 cases, 6.9%) compared to fertile subjects (5 of 323, 1.5%; Fisher’s exact test: odds ratio (OR) = 4.7, 95% CI 1.8–15.5; *P* = 2.3 × 10^−4^). The prevalence of findings was not significantly different between azoospermic and oligozoospermic cases. There was also no enrichment of findings in men with a history of cryptorchidism (Table 3).

**Table 3.** Distribution of findings in hereditary cancer genes in the ESTAND cohort study subgroups.

Compared study subjects		Hereditary cancer-linked findings	Fisher’s exact test	
Subgroup 1	Subgroup 2	Carriers (n, %)	OR [95% CI]	*P*-value^a^
Infertile	Fertile	36/522 (6.9%) vs 5/323 (1.5%)	4.7 [1.8–15.5]	2.3 × 10^–4^
NOA	Fertile	19/280 (6.8%) vs 5/323 (1.5%)	4.6 [1.6–16.1]	1.3 × 10^–3^
Oligozoosp.	Fertile	17/242 (7.0%) vs 5/323 (1.5%)	4.8 [1.7–17.0]	1.4 × 10^–3^
Infertile, CR	Fertile	15/156 (10%) vs 5/323 (1.5%)	6.7 [2.3–24.2]	8.9 × 10^–5^
Infertile, no CR	Fertile	21/366 (5.7%) vs 5/323 (1.5%)	3.9 [1.4–13.3]	4.4 × 10^–3^
NOA	Oligozoosp.	19/280 (6.8%) vs 17/242 (7.0%)	1.0 [0.5–2.2]	ns
Infertile, CR	Infertile, no CR	15/156 (10%) vs 21/366 (5.7%)	1.8 [0.8–3.7]	ns

a Statistical significance between subgroups was assessed using Fisher’s exact test.

Non-obstructive azoospermia (NOA) indicates a complete lack of sperm, whereas oligozoospermia (Oligozoosp.) is defined as total sperm count ≤39 million per ejaculate (World Health Organization, 2021). Cryptorchidism (CR) refers to at least one testicle missing in the scrotum at the recruitment or medical history of CR resolved by orchidopexy or spontaneous descent.

ESTAND, ESTonian ANDrology; OR, odds ratio.

### Overrepresentation of findings in DNA repair genes

The proportion of analyzed genes with findings in each molecular subgroup was broadly similar (18–23%; Fig. 1A). However, DNA repair genes stood out for the total load of LP/P variants (26 of 41 men with findings; 63%; Fig. 1B). Among these, affected Fanconi anemia (FA) pathway genes accounted for 20 subjects (17 infertile and three fertile). FA-pathway genes *BRCA2* (five different heterozygous cases) and *FANCM* (five cases with recurrent biallelic LoFs) were the most frequently affected loci (Table 2). One NOA patient with bilateral cryptorchidism and severe hypospadias carried two previously reported truncating variants in FA genes, BRIP1 p.Thr912fs (Breast Cancer Association Consortium, 2021) and *RAD51C* c.1026 + 5_1026 + 7del (Loveday *et al.*, 2012).

### Disease-causing variants in pleiotropic genes linked to syndromic phenotypes

The patient (case 37) carrying a previously unreported variant TSC1 p.Ala428fs presented clinical symptoms compatible with tuberous sclerosis, such as hypomelanotic macules and shagreen patches, facial angiofibroma and ungual fibroma, renal cysts, idiopathic kidney failure, and childhood-onset epilepsy (Table 2). His mother had also suffered from idiopathic epilepsy, and his maternal grandmother had been diagnosed with renal cancer.

NOA patient (case 31) with PHOX2B p.Phe33fs variant had been diagnosed with Hirschsprung disease and ileus, neoplasms of unclear nature, and unilateral cryptorchidism. Both NOA subjects (cases 39 and 40) with *WT1* missense substitutions (p.Met415Lys, p.Trp151Gly) had congenital renal conditions. Case 40 had also been diagnosed with basalioma and presented a history of cancer in the family. LP/P variants in *MLH1*, *MSH2*, and *MSH6* linked to Lynch syndrome (hereditary nonpolyposis colorectal cancer) were observed in four infertile men with a history of cryptorchidism. During infertility workup at the age of 26–33 years, these patients had not developed Lynch syndrome.

Three patients in the ESTAND cohort had findings in pleiotropic genes *SPRED1*, *NF1*, and *LZTR1* linked to developmental syndromes referred to as RASopathies, including various cancerous and non-cancerous tumors as part of the respective syndromic phenotype (Juchnewitsch *et al.*, 2024). Case 23, who carried LZTR1 p.Arg283Gln, had been diagnosed with schwannomatosis, a rare tumor of the cranial nerve. Case 28 presented with characteristic features of neurofibromatosis 1, carried NF1 p.Ala1450Ser, and had a family history of early-onset breast cancer. Case 35 with digenic findings SPRED1 p.Arg325Ter and TP63 p.Pro428Leu presented characteristic features of Legius syndrome (linked to *SPRED1*). His family members had been diagnosed with colon and cervical cancer.

Three patients carried LP variants in *HOXB13*, primarily linked to prostate cancer (Economides and Capecchi, 2003), including p.Gly84Glu substitution found in two NOA cases. One of them had a medical history of unilateral cryptorchidism and Klippel–Trenaunay–Weber syndrome.

### History of cancer in men with findings and their families

Retrospective medical records were available for 36 out of 41 (88%) men with LP/P findings (Table 2, Supplementary Table S6). Six infertile men presented a history of cancerous or non-cancerous tumors—leiomyosarcoma (BRCA2 p.Ser599Ter), lymphoma (TP53 p.Arg181His), basalioma (WT1 p.Trp151Gly), fibroma and renal cysts (TSC1 p.Ala428fs), neoplasms of uncertain behavior (PHOX2B p.Phe33fs), and schwannomatosis (LZTR1 p.Arg283Gln) (Table 2).

A family history of cancer had been documented for 10 of 14 infertile men with available pedigree health data (Table 2, Supplementary Table S6). On most occasions, the reported tumor types matched the patient’s genetic findings, e.g. early-onset breast (family history of patients with findings in *BRCA1*, *BRCA2*, *BRIP1*, *NF1*), prostate (*BRCA2*, *FANCM*, *TP53*, *WT1*), renal (*TSC1*), gynecological (*BRIP1*, *TSC1*), and hematological cancers (*MLH1*, *TP53*, *WT1*). Family members were not available for cascade screening and segregation analysis in the pedigree.

### Testis expression and mouse models suggest shared genetics of cancer and male infertility

Two-thirds of hereditary cancer genes with LP/P findings (16 of 24) are highly expressed in one or more human testicular cell types, including specific stages of spermatogenesis (Table 4).

**Table 4.** Hereditary cancer genes with (likely) pathogenic findings and their reported contribution to spermatogenesis and predisposition to cancer.

	LP/P variants in this study	Characteristics of the human gene		Mutant mouse models for the gene^b^	
Gene name	Number of LoF/missense	Functional category	Male-specific tissue expression^a^	Abnormal male reproductive phenotype	Predisposition to cancer
*ATM*	0/1	DNA repair	Increased expression in male-specific tissues not reported	Infertile, small testes (hom)	Yes (het/hom)
*BARD1*	1/0	DNA repair	Spermatogonia, spermatocytes	Male infertility, small testes (hom)	Yes (conditional)
*BRCA1*	2/1	DNA repair	Spermatocytes*, spermatogonia*	Infertile, small testes (hom)	Yes (hom/het)
*BRCA2*	4/1	DNA repair	Spermatocytes*, spermatogonia*	Infertile, small testes (hom)	Yes (hom/het)
*BRIP1*	2/1	DNA repair	Spermatids*	Subfertile, small testes (hom)	Yes (hom)
*CHEK2*	0/1	Tumor suppressor	Spermatogonia*	None reported	Yes (hom/het)^§^
*EGFR*	1/0	Development	Increased expression in male-specific tissues not reported	None reported	Yes (transgenic)
*FANCM*	3/0	DNA repair	Spermatids*	Infertile, small testes (hom)	Yes (hom/het)
*HOXB13*	0/2	Development	Prostate	Abnormal prostate gland (hom)	Yes (transgenic)
*LZTR1*	0/1	Tumor suppressor	Prostate	None reported	Yes (transgenic)
*MLH1*	0/1	DNA repair	Spermatocytes, spermatids	Infertile, small testes (hom)	Yes (hom/het)
*MSH2*	0/2	DNA repair	Spermatocytes, spermatogonia	None reported	Yes (hom/het)
*MSH6*	1/0	DNA repair	Spermatids, spermatogonia, spermatocytes	None reported	Yes (hom/het)
*NF1*	0/1	Tumor suppressor	Spermatids	Subfertile, cryptorchidism^#^ (het)	Yes (het)
*PALB2*	2/0	DNA repair	Spermatocytes*, spermatogonia	Subfertile, small testes (hom)	Yes (transgenic)
*PHOX2B*	1/0	Development	Increased expression in male-specific tissues not reported; enriched in the adrenal gland	None reported	Yes (transgenic)
*RAD51C*	3/0	DNA repair	Spermatocytes*, spermatids*	Subfertile (het)	Yes (conditional)
*SMAD4*	0/1	Tumor suppressor	Prostate	Infertile, small testes (transgenic)	Yes (het)
*SPRED1*	1/0	Development	Spermatid	Abnormal seminal vesicle morphology (hom)	Yes (transgenic)
*TP53*	0/1	Tumor suppressor	Increased expression in male-specific tissues not reported	Abnormal spermatogenesis, small testes (hom)	Yes (hom/het)
*TP63*	0/1	Development	Increased expression in male-specific tissues not reported	Absent prostate gland (hom)	Yes (het)
*TSC1*	1/0	Tumor suppressor	Spermatids*	Abnormal spermatogenesis, small testes (conditional)	Yes (het)
*TSC2*	0/1	Tumor suppressor	Prostate, spermatogonia	Abnormal spermatogenesis, small testes (conditional)	Yes (het)
*WT1*	0/2	Development	Sertoli cells*	Infertile, small testes (hom/het)	Yes (conditional)

a Expression data based on The Human Protein Atlas (https://www.proteinatlas.org/) with the highest levels of expression listed first. Cell-type enhancement, showing a specialized role in spermatogenesis, is marked with the symbol *.

b Reported mouse models with reproductive male phenotype and predisposition to cancer were based on the Mouse Genome Informatics database (https://www.informatics.jax.org/) and published literature (Supplementary Table S7). Most of the data are from homozygous (hom) or heterozygous (het) knockout mouse models, except when indicated otherwise (conditional or transgenic).

§ Predisposition to cancer shown in mutant mice with CHEK2*1100delC variant.

# Missense substitution p.Asn1453Lys introduced to the mouse model is localized close to the variant p.Ala1450Ser identified in ESTAND case 28.

LP, likely pathogenic; LoF, loss-of-function; P, pathogenic.

Mutant mouse models for *Atm*, *Bard1*, *Brca1*, *Brca2*, *Brip1*, *Fancm*, *Mlh1*, *Nf1*, *Palb2*, *Rad51C*, *Smad4*, *Tp53*, *Tsc1*, *Tsc2*, and *Wt1* have been reported to exhibit male sub- or infertility, abnormal male meiosis, congenital gonadal dysgenesis, and/or gonadal atrophy. Reproductive phenotype was primarily observed in homozygous mutant mice except for *Nf1*, *Rad51C*, and *Wt1*, whereby heterozygous mutant males present the sub- or infertility. Mutant mouse models of all 24 genes have shown predisposition to various tumors. On most occasions, the risk of cancer was already observed in heterozygous animals (Table 4, Fig. 1C, Supplementary Table S7).

### *CHEK2* cancer risk variants are not enriched in infertile men

Additionally, we analyzed known risk variants for cancer susceptibility in Central and Eastern Europe, CHEK2 p.Ile157Thr (population prevalence in Estonia 8.6%), CHEK2 p.Thr367fs (0.6%), and *CHEK2*: c.319 + 2T>A (0.1%) (Pavlovica *et al.*, 2022). There were no significant differences in allele frequencies of these variants between infertile and fertile men (Supplementary Table S8). Likewise, allele frequencies of the ESTAND patient and control groups did not differ statistically from the data reported in the population-based Estonian Biobank study (Pavlovica *et al.*, 2022).

## Discussion

This study showed almost a 5-fold enrichment of disease-causing findings in hereditary cancer genes in infertile compared to fertile men (6.9% vs 1.5%, *P* = 2.3 × 10^−4^) (Tables 2 and 3). One in 15 azoo- or oligozoospermic patients was identified as a carrier of LP/P variants linked to monogenic hereditary cancers. Several of these men had been diagnosed with cancer by the time of the study, typically in early adulthood. Notably, one or more incidences of cancer among family members had been documented for 10 (of 14) infertile men with available pedigree health data.

The data from this study align well with epidemiological research, showing that men with lower sperm counts have a 2-fold higher prevalence of cancer compared to the general population (Eisenberg *et al.*, 2013; Ramsay *et al.*, 2024). Consistently, our recent study observed a 4-fold increased incidence of various cancer types in men with monogenic infertility (median age at recruitment: 32 years) compared to 40- to 49-year-old men representing the general Estonian population (Lillepea *et al.*, 2024). Hereditary cancer is diagnosed at a median age of ∼40 years (Sosinsky *et al.*, 2024). Therefore, the true incidence of cancer among our study subjects with findings in the hereditary cancer gene panel (median 32 years) was not possible to estimate.

Epidemiological studies have also reported that azoospermia cases have a more than 2-fold higher risk of cancers than other forms of male infertility (Eisenberg *et al.*, 2013). In our study, the observed burden of hereditary cancer-linked variants was not statistically different in azoospermia compared to oligozoospermia patients and in men with or without a history of cryptorchidism (Table 3).

Shared molecular etiology of cancer and spermatogenic failure has been discussed as both conditions involve impaired DNA repair and genome integrity, cellular proliferation, and differentiation. Some hereditary cancer genes (e.g. *BRCA2*, *FANCM*, *MLH1*, *WT1*) have been directly linked to human infertility (Zhoucun *et al.*, 2006; Ji *et al.*, 2012; Xu *et al.*, 2017; Kasak *et al.*, 2018). While monoallelic disease-causing variants are typically sufficient for the development of cancer, carriership of biallelic variants is usually needed to cause infertility. However, it is possible that heterozygous mutations in cancer-linked genes may still co-contribute to reproductive phenotype, as mild forms of subfertility may be overlooked or are challenging to distinguish from iatrogenic causes in cancer patients. This scenario is consistent with several respective mutant mouse models presenting sub- or infertility (Table 4, Fig. 1C).

Half of the infertile men were identified with LP/P variants in DNA repair genes, including a high number of findings in the FA pathway (17 of 36 infertile men with LP/P variants) with a critical role in DNA replication, repair, recombination, and maintenance of genome stability in mitosis and meiosis (Fig. 1B). Most of these genes exhibit high expression in specific testicular cell types (Table 4). The affected FA pathway has been linked to both impaired spermatogenesis and cancer predisposition (Peake and Noguchi, 2022). The inability to correct DNA errors during numerous mitotic cycles and defects in the complex recombination process may predispose to male infertility.

Some patients had LP/P variants in genes linked not only to hereditary cancers but also to syndromic developmental conditions with a broader phenotype, such as tuberous sclerosis (*TSC1*) (Randle, 2017), Hirschsprung disease (*PHOX2B*) (Fernández *et al.*, 2013), congenital anomalies of the kidney (*WT1*) (Lopez-Gonzalez and Ariceta, 2024), and RASopathies such as Legius syndrome (*SPERD1*), Noonan syndrome (*LZTR1*), and neurofibromatosis 1 (*NF1*) (Juchnewitsch *et al.*, 2024). The gathered health records were consistent with these findings in pleiotropic genes contributing to the development and function of multiple tissues and organs, including the genitourinary system.

As an interesting observation, four infertile men with cryptorchidism had disease-causing findings in *MLH1*, *MSH2*, or *MSH6* linked to Lynch syndrome. To the best of our knowledge, no previous study has reported testicular maldescent in Lynch syndrome patients, and respective knockout mouse models do not present affected testicular development (Baker *et al.*, 1996; Klonisch *et al.*, 2004; He *et al.*, 2012). Therefore, further research is needed to clarify whether this observation was a chance finding or indicates an overlapping genetic etiology. These patients (ages 26–33 years) did not have a medical history of cancer at recruitment. However, the risk of developing Lynch syndrome varies depending on the specific gene and the individual’s sex, ranging from 30% to 80%, with a median age of onset above 45 years (Peltomäki *et al.*, 2023).

Despite the carriership of a cancer-linked genetic variant not always leading to tumor development, timely screening and counseling will have immediate clinical benefits. In a clinical setting, management of infertility typically occurs at a younger age than the progression and diagnosis of cancer. Therefore, early identification of a genetic predisposition to cancer is critical for optimal and effective patient monitoring and, if needed, early intervention. Moreover, since some hereditary cancer syndromes tend disproportionately to affect female family members (e.g. *BRCA1*, *BRCA2*, *PALB2* variants and breast cancer; National Comprehensive Cancer Network, 2024), Lynch syndrome and endometrial cancer (Lu and Broaddus, 2020), cascade screening among family members will offer apparent clinical benefits.

In summary, the current study showed a high prevalence of hereditary cancer-linked findings among infertile men, supporting shared monogenic etiologies of cancer and spermatogenic failure. This might explain, at least partially, the higher prevalence of cancer reported in infertile compared to fertile men in epidemiological studies (Eisenberg *et al.*, 2015). The study has immediate clinical implications as men typically seek infertility management in their 30s when they are asymptomatic for cancer on most occasions. Inclusion of hereditary cancer genes in the recently proposed clinical exome of infertile men (Lillepea *et al.*, 2024) will provide a significant added value, enabling timely counseling and management of reproductive and general health strategies.

## Supplementary Material

hoaf008_Supplementary_Data

## Data Availability

The data underlying this article are available in the article and in its online supplementary material. All hereditary cancer-linked variants identified in this study have been submitted to the NCBI ClinVar database (https://www.ncbi.nlm.nih.gov/clinvar/).
