# Supplementary material for: Significantly increased load of hereditary cancer-linked germline variants in infertile men
Source: Hum Reprod Open. 2025 Feb 21;2025(2):hoaf008. doi: 10.1093/hropen/hoaf008 (PMC11889456; doi:10.1093/hropen/hoaf008)
Supplement: hoaf008_Supplementary_Data [file hoaf008_supplementary_data.zip › Supplementary-materials-and-methods-post adjudication clean.docx]

**Supplementary Materials and Methods**

An overview of the study design and flow is shown in **Supplementary Fig S1**.

**Subject recruitment, clinical phenotyping**

All 845 study participants were recruited at the Andrology Clinic of Tartu University Hospital (AC-TUH), the primary and referral center managing >90% of all male infertility cases in Estonia. AC-TUH is an accredited member of the European Academy of Andrology (EAA), and all andrologists have received training in standardized clinical assessment in the EAA centers.

All men received identical routine andrological workups by andrology specialists using the established clinical pipeline and standard protocols at the AC-TUH (Punab *et al.*, 2017; World Health Organization, 2021). Physical examination included the assessment of genital phenotype, testicular size (evaluated with an orchidometer by birch wood, Pharmacia & Upjohn, Denmark) and position, pathologies of the genital duct, urethra, and presence of varicocele. Reduced total testis volume was defined as <30 ml. Sperm samples were collected according to the recommendations of the WHO (World Health Organization, 2021) acquired by masturbation. The semen samples were incubated at 37°C for 30-40 minutes for liquefaction. Then, the volume was estimated by weight, subtracting the collection tube weight and assuming 1 g = 1 ml. The concentration of spermatozoa was estimated on diluted samples (with 0.6 mol/l NaHCO_3_%, 0.4% (v/v) formaldehyde in distilled water) using the improved Neubauer hemocytometers.

Additionally, blood samples were taken for reproductive hormonal analysis between 8 a.m. and 10:30 a.m. on the same day the semen sample was produced. Blood was centrifuged, and serum was used to determine testosterone, follicle-stimulating hormone (FSH), and luteinizing hormone (LH) by electrochemiluminescence immunoassay (ECLIA) method (Cobas e 601, Roche Diagnostics). All analyses were carried out at the United Laboratories, Tartu University Hospital, and the reference ranges are provided in **Supplementary Table S1**.

**Formation of the candidate gene list**

The analyzed panel of 157 genes linked to hereditary cancer syndromes comprised 113 genes in the TruSight Hereditary Cancer Panel (“TruSight Hereditary Cancer Panel | Used with Nextera Flex for Enrichment,” n.d.), ten additional genes reported in the OMIM database and 34 genes reported in the COSMIC Cancer Gene Census (https://cancer.sanger.ac.uk/census) with a clear hereditary cancer type or syndrome (**Supplementary Table S2**). Genes were stratified based on the disease inheritance mode according to the Illumina TruSight Hereditary Cancer Panel, COSMIC Cancer Gene Census data, OMIM database, or Genomics England PanelApp (https://panelapp.genomicsengland.co.uk/). Most genes were linked to autosomal dominant forms of hereditary cancers (AD, 85/157, 54%), followed by autosomal recessive (AR 45/157, 29%), AD/AR genes depending on the phenotype (23/157, 15%) and only four X-linked genes (XL, 3%).

**WES data generation, processing of VCF files, variant annotation using Variant Effect Predictor**

A total of 522 men with infertility and 323 fertile men were subjected to whole exome sequencing (WES) using genomic DNA extracted from blood samples (Juchnewitsch *et al.*, 2024; Lillepea *et al.*, 2024). NGS library preparation, sequencing, primary sequence analysis, and variant calling were performed in three sequencing centers: 448 DNA samples were sequenced at the Next Generation Sequencing Service laboratory of the Institute for Molecular Medicine Finland (FIMM), Helsinki, Finland; 82 DNA samples at the McDonnell Genome Institute of Washington University in St. Louis, MO, USA and 315 at the Huntsman Cancer Institute High-Throughput Genomics Core Facility at the University of Utah in Salt Lake City, UT, USA and were delivered as VCF files. Details of WES data generation can be found in (Juchnewitsch *et al.*, 2024; Lillepea *et al.*, 2024).

Individual VCF files generated in all sequencing service centers were filtered for quality using identical parameters. Variants with low depth of coverage (DP<10) and low genotype quality (GQ<20) were excluded. Individual samples were further processed by filtering out heterozygous variants in the non-PAR regions of chromosome X (GRCh38 assembly, chrX:1-10000; chrX:2781480-155701382; chrX:156030896-156040895) and chromosome Y (chrY:1-10000; chrY:2781480-56887902; chrY:57217416-57227415). After quality control, all individual filtered VCF files were merged into a single VCF file and then segmented into individual chromosome files (n=24: chr1-22, X, and Y) for variant annotation. Merging, filtering, and splitting of VCF files was performed with bcftools (v1.14) (Danecek *et al.*, 2021). The individual chromosome VCF files (n=24) were annotated with Ensembl Variant Effect Predictor (VEP; v105) (McLaren *et al.*, 2016) in the offline mode using the set of flags and plugins listed in **Supplementary Tables S3, S4**.

**Filtering and prioritization of variants in candidate genes linked to hereditary cancers**

The study focused on discovering monogenic likely pathogenic and pathogenic (LP/P) variants in the hereditary cancer genes, i.e., diagnostic DNA variants with known or confidently predicted causal link to the cancer. Most hereditary cancer-linked variants are rare in the general population but have a large individual effect on cancer development. VEP output files were subjected to a custom-designed pipeline to automatically exclude variants with an unlikely disease-causing effect (**Supplementary Table S3, S4**). Variants reported as confidently pathogenic (P) and/or likely pathogenic (LP) in the NCBI ClinVar database (Landrum *et al.*, 2018) were automatically retrieved from the VEP output file, column 'clinvar_clnsig'. For the rest of the variants, (i) minor allele frequency (MAF) <0.5% in the general population and (ii) Combined Annotation Dependent Depletion (CADD) score ≥20 were used as cutoffs to include rare variants with possible disease-causing effect. CADD score is a summary statistic for scoring the deleteriousness of single nucleotide variants, combining predictions of many alternative algorithms (Rentzsch *et al.*, 2019) (https://cadd.gs.washington.edu). gnomAD database (version 4.0.0, <https://gnomad.broadinstitute.org>) was used as the source for the observed MAF of identified variants across global populations and specifically in Europe. Genes linked to AR cancer phenotypes with singleton heterozygous variants per subject were excluded from further assessment.

The interpretation of retained variants was facilitated by the online AI-based platform Franklin by Genoox (<https://franklin.genoox.com>), which is an openly available source to process and annotate NGS data, providing additional information and assessment of variants and genes. In addition, ClinVar (Landrum *et al.*, 2018) submissions of retained variants were inspected. Variants unanimously predicted to be benign or likely benign based on the two sources were excluded. To confirm, all retained variants passed a visual inspection of the quality of sequencing reads using the Integrative Genomics Viewer (IGV) software (Robinson *et al.*, 2023), and variants with low confidence were discarded. All remaining variants underwent manual pathogenicity assessment according to the American College of Medical Genetics and Genomics (ACMG) guidelines (Richards *et al.*, 2015), and the final assessment took into account *in silico* predictions, previous literature, and additional retrospective clinical data available to researchers, which was done by two researchers.

All variants predicted to be disease-causing were further validated by Sanger sequencing. PCR primers are available in **Supplementary Table S5**, and chromatograms of all validated variants are shown in **Supplementary Fig 2**. Variant MSH6 p.Asp857fs (case 27) was confirmed in a clinical-grade accredited molecular diagnostics laboratory, the United Laboratory at Tartu University Hospital.

**Collecting retrospective clinical and family health data in regard to cancer diagnosis**

For patients with findings in the hereditary cancer genes, a retrospective assessment of available clinical data was performed with a focus on cancer. General health data was available for 36 of 41 (88%) men, and family health history for 14 of 41 (34%) men with findings (**Supplementary Table S6**).

**References**

Danecek P, Bonfield JK, Liddle J, Marshall J, Ohan V, Pollard MO, Whitwham A, Keane T, McCarthy SA, Davies RM, *et al.* Twelve years of SAMtools and BCFtools. *GigaScience* 2021;**10**:giab008.

Juchnewitsch A-G, Pomm K, Dutta A, Tamp E, Valkna A, Lillepea K, Mahyari E, Tjagur S, Belova G, Kübarsepp V, *et al.* Undiagnosed RASopathies in infertile men. *Front Endocrinol* 2024;**15**:1312357.

Kasak L, Lillepea K, Nagirnaja L, Aston KI, Schlegel PN, Gonçalves J, Carvalho F, Moreno-Mendoza D, Almstrup K, Eisenberg ML, *et al.* Actionable secondary findings following exome sequencing of 836 non-obstructive azoospermia cases and their value in patient management. *Hum Reprod* 2022;**37**:1652–1663.

Kasak L, Punab M, Nagirnaja L, Grigorova M, Minajeva A, Lopes AM, Punab AM, Aston KI, Carvalho F, Laasik E, *et al.* Bi-allelic Recessive Loss-of-Function Variants in FANCM Cause Non-obstructive Azoospermia. *Am J Hum Genet* 2018;**103**:200–212.

Landrum MJ, Lee JM, Benson M, Brown GR, Chao C, Chitipiralla S, Gu B, Hart J, Hoffman D, Jang W, *et al.* ClinVar: Improving access to variant interpretations and supporting evidence. *Nucleic Acids Res* 2018;**46**:D1062–D1067.

Lillepea K, Juchnewitsch A-G, Kasak L, Valkna A, Dutta A, Pomm K, Poolamets O, Nagirnaja L, Tamp E, Mahyari E, *et al.* Toward clinical exomes in diagnostics and management of male infertility. *Am J Hum Genet* 2024;**111**:877–895.

McLaren W, Gil L, Hunt SE, Riat HS, Ritchie GRS, Thormann A, Flicek P, Cunningham F. The Ensembl Variant Effect Predictor. *Genome Biol* 2016;**17**:122.

Punab M, Poolamets O, Paju P, Vihljajev V, Pomm K, Ladva R, Korrovits P, Laan M. Causes of male infertility: a 9-year prospective monocentre study on 1737 patients with reduced total sperm counts. *Hum Reprod* 2017;**32**:18–31.

Rentzsch P, Witten D, Cooper GM, Shendure J, Kircher M. CADD: predicting the deleteriousness of variants throughout the human genome. *Nucleic Acids Res* 2019;**47**:D886–D894.

Richards S, Aziz N, Bale S, Bick D, Das S, Gastier-Foster J, Grody WW, Hegde M, Lyon E, Spector E, *et al.* Standards and guidelines for the interpretation of sequence variants: a joint consensus recommendation of the American College of Medical Genetics and Genomics and the Association for Molecular Pathology. *Genet Med Off J Am Coll Med Genet* 2015;**17**:405–424.

Robinson JT, Thorvaldsdóttir H, Turner D, Mesirov JP. igv.js: an embeddable JavaScript implementation of the Integrative Genomics Viewer (IGV). In Alkan C, editor. *Bioinformatics* 2023;**39**:btac830.

TruSight Hereditary Cancer Panel | Used with Nextera Flex for EnrichmentAvailable from: https://www.illumina.com/products/by-type/clinical-research-products/trusight-cancer-hereditary.html.

World Health Organization. WHO laboratory manual for the examination and processing of human semen Sixth Edition 2021.
