# Supplementary material for: Significantly increased load of hereditary cancer-linked germline variants in infertile men
Source: Hum Reprod Open. 2025 Feb 21;2025(2):hoaf008. doi: 10.1093/hropen/hoaf008 (PMC11889456; doi:10.1093/hropen/hoaf008)
Supplement: hoaf008_Supplementary_Data [file hoaf008_supplementary_data.zip › Figure-S2-post_adjudication_clean.docx]

**
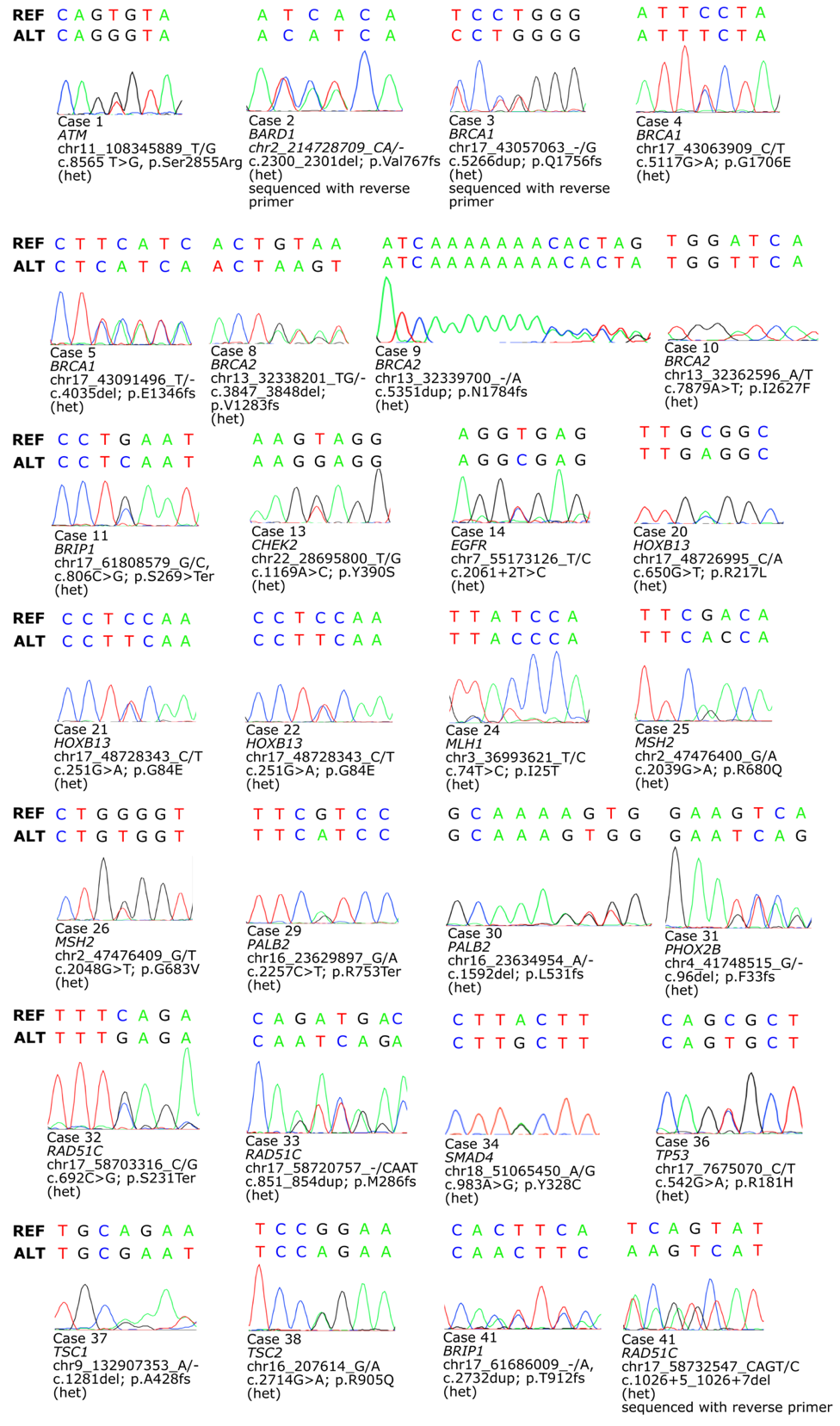
**

**Supplementary Figure S2. Chromatograms of variant validation by Sanger sequencing.** Chromatograms are shown for variants first mentioned within this article. Chromatograms for previously reported variants are available in the original papers (Kasak *et al.*, 2018, 2022; Juchnewitsch *et al.*, 2024; Lillepea *et al.*, 2024). Variants are listed in the order of case number. Primers for PCR and sequencing are presented in **Supplementary Table S5.**

ALT, alternative; het, heterozygous; REF, reference.
