# Supplementary material for: Significantly increased load of hereditary cancer-linked germline variants in infertile men
Source: Hum Reprod Open. 2025 Feb 21;2025(2):hoaf008. doi: 10.1093/hropen/hoaf008 (PMC11889456; doi:10.1093/hropen/hoaf008)
Supplement: hoaf008_Supplementary_Data [file hoaf008_supplementary_data.zip › Figure-S1-post_adjudication_clean.docx]

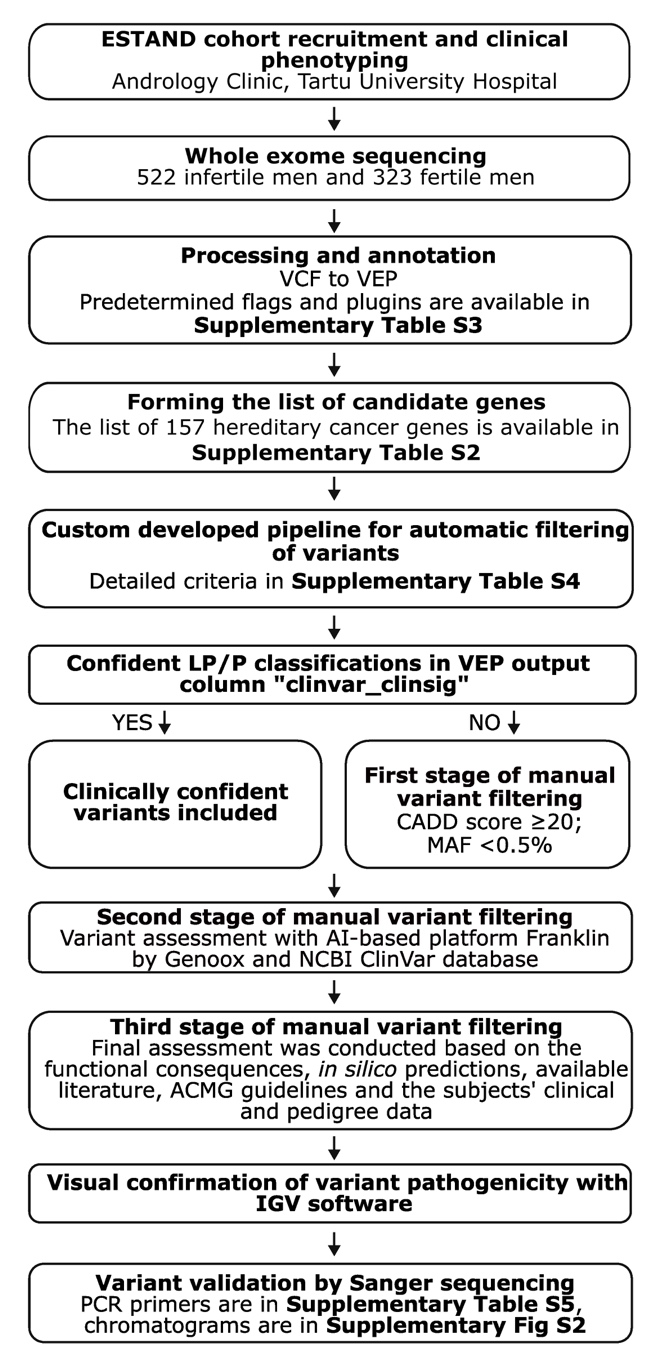


**Supplementary Figure S1. Study design and flow of data collection and analysis.**

Variant pathogenicity evaluation used ClinVar (https://www.ncbi.nlm.nih.gov/clinvar/) database records and AI-based platform Franklin by Genoox (<https://franklin.genoox.com>). All retained variants were visualized with Integrative Genomics Viewer (IGV) (Robinson *et al.*, 2023). The final manual assessment of variants was based on the American College of Medical Genetics and Genomics (ACMG) guidelines (Richards *et al.*, 2015).

ESTAND, ESTonian ANDrology; LP, likely pathogenic; MAF, minor allele frequency; P, pathogenic; VCF, variant call format; VEP, Variant Effect Predictor.
